# Supplementary material for: Vitamin D Modified DSS-Induced Colitis in Mice via STING Signaling Pathway
Source: Biology (Basel). 2025 Jun 18;14(6):715. doi: 10.3390/biology14060715 (PMC12190092; doi:10.3390/biology14060715)
Supplement: Supplementary file 1 [file biology-14-00715-s001.zip › Figures S1–S5: Origin images for blots.pdf]

Supplementary Materials

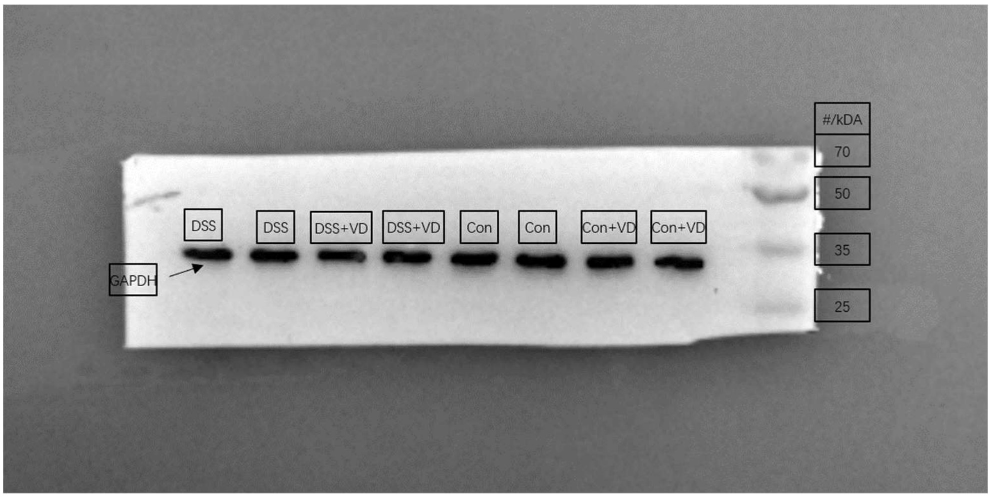

Figure S1 Western blot membrane of GAPDH (~36 kDa) protein detected with GAPDH Mouse mAb (High Dilution)(AC033; 1:100000; ABclonal, China) antibody. Gel-separated proteins were transferred to Immobilon® -PSQ PVDF Membrane (0.2 µm pore size; ISEQ00010; Merck KGaA, Darmstadt, Germany) by wet transfer method (250mA, 60Min). Membranes, incubated with a horseradish peroxidase-conjugated secondary antibody (ab205718; 1:20000; Abcam), were developed with BeyoECL Star(Beyotime, China). #Weight marker (molecular weight in kDa): Thermo Scientific™/PageRuler™ Prestained Protein Ladder, 10 to 180 kDa; catalogue number: 26616.Blot images, prior to the densitometry readings, were converted to grayscale with ImageJ (ImageJ v.1.54g, National Institutes of Health, Maryland, USA) as follows: Image -> Type –> 8 bit, next: Image -> Adjust–> Brightness/Contrast –> Auto. DSS- the DSS-induced colitis group; DSS+VD- the vitamin D treatment DSS-colitis group; Con-the control group; Con+VD- the vitamin D treatment group.

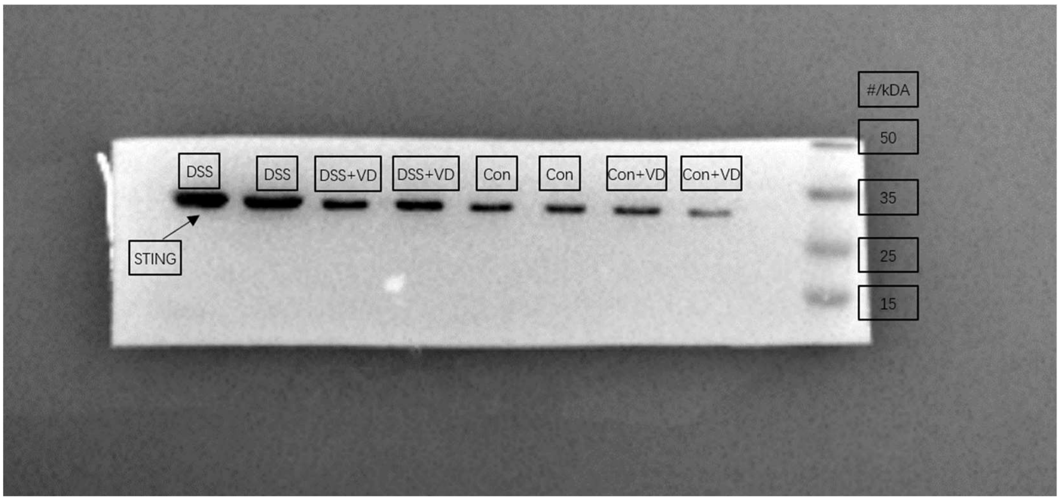

Figure S2 Western blot membrane of STING (33~35 kDa) protein detected with Anti-STING antibody [EPR25090-107] (ab288157; 1:1000; Abcam). Gel-separated proteins were transferred to Immobilon® -PSQ PVDF Membrane (0.2 µm pore size; ISEQ00010; Merck KGaA, Darmstadt, Germany) by wet transfer method (250mA, 60Min). Membranes, incubated with a horseradish peroxidase-conjugated secondary antibody (ab205718; 1:20000; Abcam), were developed with BeyoECL Star (Beyotime, China). #Weight marker (molecular weight in kDa): Thermo Scientific™/PageRuler™ Prestained Protein Ladder, 10 to 180 kDa; catalogue number: 26616.Blot

images, prior to the densitometry readings, were converted to grayscale with ImageJ (ImageJ v.1.54g, National Institutes of Health, Maryland, USA) as follows: Image -> Type –> 8 bit, next: Image -> Adjust–> Brightness/Contrast –> Auto. DSS- the DSS-induced colitis group; DSS+VD- the vitamin D treatment DSS-colitis group; Con-the control group; Con+VD- the vitamin D treatment group.

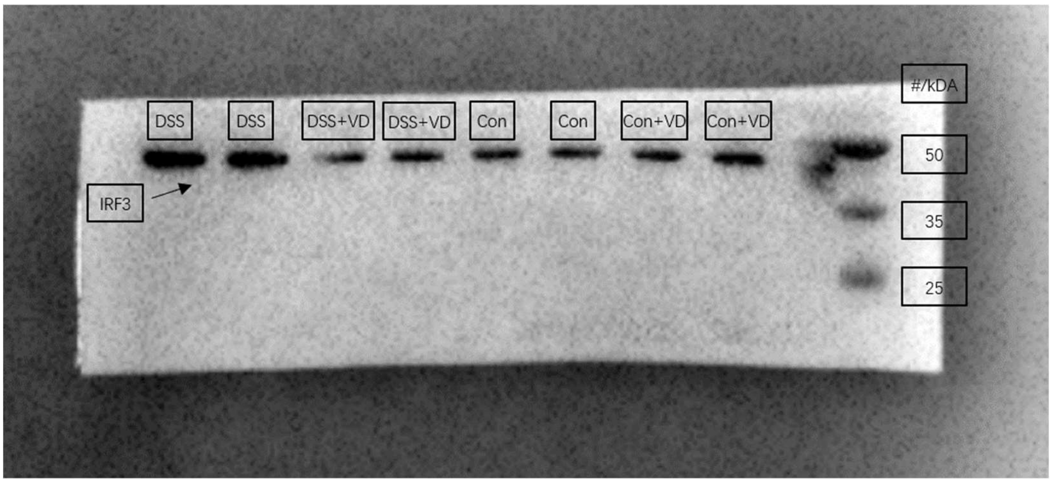

Figure S3 Western blot membrane of IRF3 (45~55 kDa) protein detected with IRF3 Rabbit pAb (A2172; 1:800; ABclonal, China). Gel-separated proteins were transferred to Immobilon® -PSQ PVDF Membrane (0.2 μm pore size; ISEQ00010; Merck KGaA, Darmstadt, Germany) by wet transfer method (250mA, 60Min). Membranes, incubated with a horseradish peroxidase-conjugated secondary antibody (ab205718; 1:20000; Abcam), were developed with BeyoECL Star (Beyotime, China). #Weight marker (molecular weight in kDa): Thermo Scientific™/PageRuler™ Prestained Protein Ladder, 10 to 180 kDa; catalogue number: 26616.Blot images, prior to the densitometry readings, were converted to grayscale with ImageJ (ImageJ v.1.54g, National Institutes of Health, Maryland, USA) as follows: Image -> Type –> 8 bit, next: Image -> Adjust–> Brightness/Contrast –> Auto. DSS- the DSS-induced colitis group; DSS+VD- the vitamin D treatment DSS-colitis group; Con-the control group; Con+VD- the vitamin D treatment group.

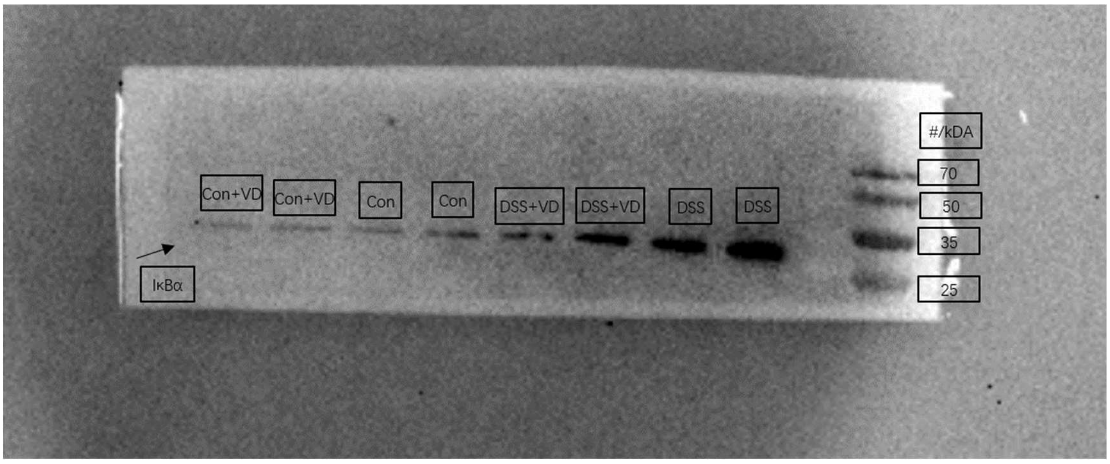

Figure S4 Western blot membrane of IκBα(~32 kDa) protein detected with IκBα Rabbit pAb (A11165; 1:1000; ABclonal, China). Gel-separated proteins were transferred to Immobilon® -PSQ PVDF Membrane (0.2 μm pore size; ISEQ00010; Merck KGaA, Darmstadt, Germany) by wet transfer method (250mA, 60Min). Membranes, incubated with a horseradish peroxidase-conjugated

secondary antibody (ab205718; 1:20000; Abcam), were developed with BeyoECL Star (Beyotime, China). #Weight marker (molecular weight in kDa): Thermo Scientific™/PageRuler™ Prestained Protein Ladder, 10 to 180 kDa; catalogue number: 26616.Blot images, prior to the densitometry readings, were converted to grayscale with ImageJ (ImageJ v.1.54g, National Institutes of Health, Maryland, USA) as follows: Image -> Type –> 8 bit, next: Image -> Adjust–> Brightness/Contrast –> Auto. DSS- the DSS-induced colitis group; DSS+VD- the vitamin D treatment DSS-colitis group; Con-the control group; Con+VD- the vitamin D treatment group.

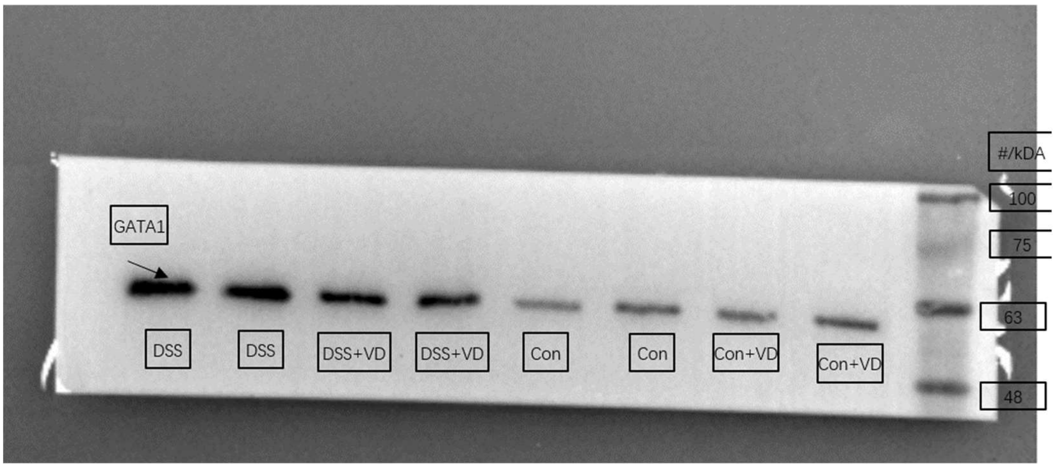

Figure S5 Western blot membrane of GATA1(~55 kDa) protein detected with anti-GATA1 Rabbit pAb [EPR26488-109] (ab300613; 1:1000; Abcam). Gel-separated proteins were transferred to Immobilon® -PSQ PVDF Membrane (0.2 µm pore size; ISEQ00010; Merck KGaA, Darmstadt, Germany) by wet transfer method (250mA, 60Min). Membranes, incubated with a horseradish peroxidase-conjugated secondary antibody (ab205718; 1:20000; Abcam), were developed with BeyoECL Star (Beyotime, China). #Weight marker (molecular weight in kDa): Solarbio ColorMixed Protein Marker(11-245KD); catalogue number PR1920. Blot images, prior to the densitometry readings, were converted to grayscale with ImageJ (ImageJ v.1.54g, National Institutes of Health, Maryland, USA) as follows: Image -> Type –> 8 bit, next: Image -> Adjust–> Brightness/Contrast –> Auto. DSS- the DSS-induced colitis group; DSS+VD- the vitamin D treatment DSS-colitis group; Con-the control group; Con+VD- the vitamin D treatment group.
